# Smart Cement-Based Materials Reinforced with CNT-Grafted CFs: Preparation and Performance Evaluation

**DOI:** 10.3390/nano15110823

**Published:** 2025-05-29

**Authors:** Xiaoyan Liu, Xiangwei Guo, Junqing Zuo, Aihua Liu, Haifeng Li, Feng Fu, Gangao Wang, Qianwen Hu, Surendra P. Shah

**Affiliations:** 1 College of Civil and Transportation Engineering, Hohai University, Nanjing 210098, China; liuaihua88@126.com; 2 College of Material Science and Engineering, Hohai University, Changzhou, 213000, China; gxw8360990@hhu.edu.cn (X.G.); m17826552691@163.com (G.W.); 15251063081@163.com (Q.H.); 3 Shanghai Construction Building Materials Technology Group Co., Ltd., Shanghai 200086, China; 4 Jiangsu Expressway Engineering Maintenance Technology Co., Ltd., Nanjing 223000, China; 5 Pearl River Water Resources Research Institute, Guangzhou 510000, China; lhfzky@163.com; 6 Shanghai Investigation, Design and Research Institute Co., Ltd., Shanghai 200335, China; fftget@163.com; 7 Center for Advanced Construction Materials, Department of Civil Engineering, University of Texas at Arlington, Arlington, TX 76019, USA; surendra.shah@uta.edu

**Keywords:** carbon nanotubes, carbon fibers, sensitivity, conductivity, smart cement-based material

## Abstract

Smart cement-based materials have the potential to monitor the health of structures. The performances of composites with various kinds of conductive fillers have been found to be sensitive and stable. However, poor dispersion of conductive fillers limits their application. This study adopted the coupling agent method to attach carbon nanotubes (CNTs) onto the surface of carbon fibers (CFs). The CNT-grafted CFs (CNT-CFs) were adopted as conductive fillers to develop a CNT-CF-incorporated cementitious composite (CNT-CF/CC). The feasibility of this approach was demonstrated through Scanning Electron Microscopy (SEM) analysis and X-ray Photoelectron Spectroscopy (XPS) analysis. The CNT-CF/CC exhibited excellent conductivity because of the introduction of CNTs compared with the CF-incorporated cementitious composite (CF/CC). The CNT-CF/CC reflected huge responses under different temperatures and moisture contents. Even under conditions of high humidity or elevated temperatures, the CNT-CF/CC demonstrated stable performance and exhibited a broad measurement range. The introduction of CNT-CFs also enhanced the mechanical properties of the composite, displaying superior piezoresistivity. The failure load for the CNT-CF/CC reached 25 kN and the maximum FCR was 24.77%. In the cyclic loading, the maximum FCR reached 20.03% when subjected to peak cyclic load at 45% of the failure load. The additional conductive pathways introduced by CNTs enhanced the conductivity and sensitivity of the composite. And the anchoring connection between CNT-CFs and the cement matrix has been identified as a primary factor enhancing the stability in performance.

## 1. Introduction

Nanomaterials have attracted considerable attention owing to their remarkable properties since they emerged in the public domain [1,2,3]. In the context of the construction industry, nanomaterials can enhance the mechanical performance of concrete [4]. Moreover, nanomaterials represented by carbon nanotubes (CNTs) exhibit excellent conductivity [5]. However, the conductivity of C30 concrete at 28 d was approximately 0.02 S/m [6]. Cement-based materials (CBMs) incorporating CNTs possess the capability to convert environmental influences into electrical signals [7]. Some scholars refer to this composite as smart cement-based materials (SCBMs), positing that it possesses the potential for monitoring the safety of buildings [8,9]. Despite the availability of numerous methods for assessing building health, many of these techniques are either prohibitively expensive or incompatible with buildings [10,11,12].

Due to the poor conductivity of cementitious materials, the electrical conductivity of the composite is predominantly determined by CNTs [13,14]. However, the dispersion of CNTs is relatively inadequate, resulting in a propensity for aggregation that considerably impacts the sensing performance of the material [3,15,16]. Some researchers have proposed the application of dispersants to effectively disperse CNTs [17,18,19]. However, due to the nanoscale of CNTs, researchers are still required to incorporate a substantial quantity of CNTs in order to achieve the desired performance, even when the dispersion is good [20,21]. Some researchers propose that by regulating the distribution of CNTs, it is feasible to improve the conductivity of composite, consequently minimizing the quantity of CNTs needed [22,23]. However, the implementation of these methods is often complex and may not sufficiently address practical requirements.

In addition to nano-conductive fillers, some larger-scale and inexpensive conductive fillers, such as carbon fibers (CFs) also have good performance. Luca Lavagna et al. [24] prepared conductive cementitious materials containing CFs oxidized by strong acids. The resistivity of the best sample was only 5 Ω·m. The integration of these conductive fillers emerges as a promising strategy to tackle this challenge [25,26]. Faezeh Azhari et al. [27] prepared conductive cementitious materials containing CFs and CNTs. The composites were quite repeatable and sensitive when the volume fractions (vol.%) of CFs and CNTs were 15 vol.% and 1 vol.%, respectively. Kim et al. [28] examined the conductivity of mortar incorporating CNTs and CFs. When the contents of CFs and CNTs were 0.2 to the weight of cement (wt.%) and 0.4 wt.%, respectively, the mortar had the lowest resistivity. Kim et al. [29] found that composite with the addition of CNTs and CFs had higher conductive stability. Despite the integration of CNTs and CFs demonstrating excellent performance at a reduced cost, this method does not effectively resolve the underlying issue of poor dispersion of CNTs. Chemical grafting can effectively connect CNTs to CFs (CNT-CFs) [30,31]. By this method, the controlled distribution of CNTs can be achieved simply by dispersing CFs. The proposed method can also partially resolve the issue of adhesion between fibers and the cement matrix [32]. It is important to note that CNT-CFs have not been extensively utilized in the preparation of smart cement-based materials. The majority of research is primarily concentrated on the mechanical properties of composites containing CNT-CFs.

Cui et al. [33] incorporated CNT-CFs into the cement matrix. The mechanical property of the composites incorporating 0.5 wt.% CNT-CFs was more excellent than the pure cement paste. The distribution of fibers was much better because of the higher steric effect. However, they did not explore the electrical properties of the composite in their work. Liu et al. [34] utilized grafting techniques to graft CNTs onto CFs for the preparation of a conductive composite, which exhibited favorable piezoresistivity under variable water contents and temperatures. Liu et al. [35] grafted CNTs onto CFs by a simple grafting technique and introduced them into the cement matrix. The composite demonstrated exceptional electrical conductivity and piezoresistive properties. But the steric effect of CNT-CFs in these two studies was not strong. Among them, it is not difficult to conclude that the steric effect of CNT-CFs determined the performance of the composites.

This study adopted the coupling agent method for the preparation of CNT-CF. The CNT-CFs produced by the coupling agent method have a stronger steric effect. In this study, the preparation process was relatively mild and safe. Scanning Electron Microscopy (SEM) and X-ray Photoelectron Spectroscopy (XPS) were utilized to analyze the feasibility of this technique and the characterization of CNT-CFs. After that, the CNT-CFs were introduced into the cement matrix. In order to illustrate the improvement in the grafting technique, a CF-incorporated cementitious composite (CF/CC) was also prepared. And the electrical properties and sensitive performance of the CNT-CF-reinforced cementitious composite (CNT-CF/CC) were evaluated.

## 2. Materials and Methods

### 2.1. Raw Materials

The cement adopted was P·O 42.5 grade cement. The length of the CFs was 5 mm, and the diameter of the CFs was 7 μm. Methylcellulose was chosen as the dispersant to ensure the distribution of the CFs in this study. Table 1 presents the major physical performance parameters of the cement used, while Table 2 illustrates the fundamental physical properties of the CFs utilized in this research. The CNTs used in this experiment were hydroxylated multi-walled carbon nanotubes (CNT-OH), sourced from Nanjing Xianfeng Nano Co., Ltd. Their fundamental physical properties are presented in Table 3. The -OH content was 5.58 wt.%.

3-aminopropyltriethoxysilane (KH550) was selected as a silane coupling agent. 1-Ethyl-3-(3-dimethylaminopropyl) carbodiimide (EDCI) and 1-Hydroxybenzotriazole (HOBt) were chosen as catalysts. N, N-dimethylformamide (DMF) was used as a solvent.

Raw materials were all added in proportion to the weight of cement (wt.%). Based on the previous work, the mixed proportions of the samples in this test are shown in Table 4. Tributyl phosphate [36] was employed as the defoaming agent, with a concentration of 0.13 wt.%. The water-to-cement ratio was 0.4. The CNT-CFs were synthesized by the coupling agent method.

### 2.2. Preparation of the CNT-CF/CC

**Figure 1:**
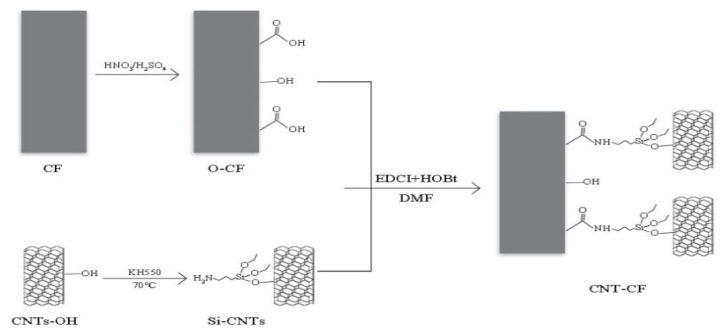
Diagram of grafting mechanism.

The grafting mechanism is illustrated in Figure 1. The acid-oxidized CFs (O-CF) had -COOH and the KH550-modified CNTs (3-aminopropyltriethoxysilane modified CNT, Si-CNT) had -NH_2_. The condensation reaction between -NH2 and -COOH connected them [37].

(1) Pre-treatment of CFs

An amount of 1 g of CFs was placed in a strong acid solution and subjected to ultrasonic dispersion for 15 min. The mixture was then allowed to undergo thorough acid oxidation at room temperature. Subsequently, the acid-treated CFs were collected, and any residual acid on their surface was thoroughly washed away. Finally, the CFs were dried in a vacuum oven for 24 h.

(2) Pre-treatment of CNTs

KH550, deionized water, and anhydrous ethanol were weighed according to a volume ratio of 1:3:6 and mixed using a stirrer. Following this step, 0.2 g of CNT-OH was added and ultrasonically dispersed for 30 min. After that, the solution was stirred in a water bath at 70 °C for 8 h to ensure a complete reaction. Upon completion of the reaction, the mixture underwent centrifugation to obtain the modified CNTs and Si-CNTs from the mixture. The Si-CNTs were repeatedly washed with anhydrous ethanol. The collected Si-CNTs were then also dried in an oven for another 24 h.

(3) Condensation reaction between CNTs and CFs

Due to the poor solubility of CNTs in water, 30 mL of DMF was poured into a beaker as a solvent. Amounts of 0.32 g of EDCI and 0.32 g of HOBt were added into the solvent as catalysts to enhance the reaction efficiency. The solution was stirred until fully dissolved to form a homogeneous solution. Next, 0.24 g of O-CFs were introduced into the solution followed by adding 0.08 g of Si-CNTs. The mixture was subjected to ultrasonic dispersion for a duration of 30 min. Subsequently, the mixture was stirred at 20 °C for 24 h. After stirring, the CNT-grafted CFs (CNT-CFs) were subjected to vacuum filtration using anhydrous ethanol for washing. Finally, the obtained CNT-CFs were dried in an oven for another 24 h.

(4) Molding method

The raw materials were prepared according to the mix proportion. Hydroxymethyl cellulose (MC) served as the dispersant for CFs. Prior to preparation, both CFs and CNT-CFs must undergo a dispersion process. The dispersion processes were as follows. Firstly, the MC was added to water at 45 °C under magnetic stirring. Subsequently, the CFs were introduced to the solution with MC and stirred magnetically for 15 min.

The cement pastes were poured into a 20 mm × 20 mm × 80 mm triple mold. Four pieces of stainless steel meshes were inserted into the paste at equal intervals. After demolding, the composites were cured in the standard environment for 28 d.

### 2.3. Test and Measurement

#### 2.3.1. Conductivity

**Figure 2:**
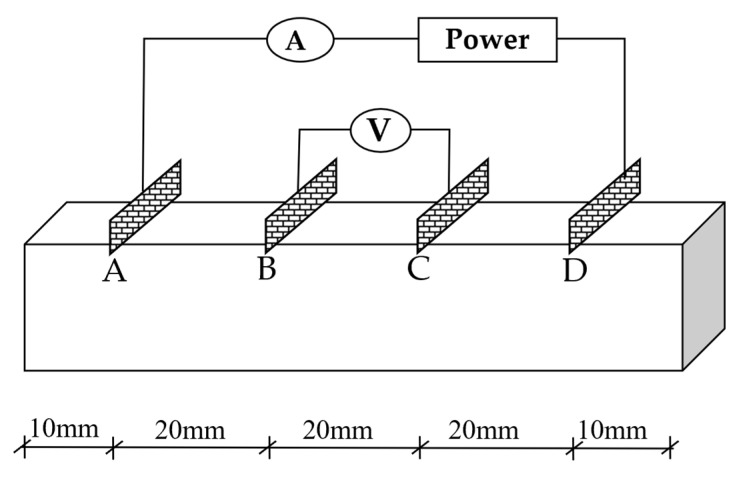
Four-electrode method.

The four-electrode method was adopted to evaluate the conductivity of composites to eliminate the influence of contact resistance. The circuit diagram is shown in Figure 2.

The external direct current (DC) power supply was constant at 10 V. The current and voltage of the specimens were measured by multimeters. After energizing for 4 to 6 min, the testing commenced.

The resistivities of the central segment (BC segment) of the specimens are considered to represent the overall resistivity of the entire sample. The resistivity is calculated as Formula (1):

(1) $$R=\frac{U}{I}$$

In the equation, R represents the resistance of the BC segment, kΩ; U represents the voltage value of the BC segment, V; and I represents the current value passing through the specimen, mA.

The formula for calculating the resistivities of the cement-based specimens can be calculated according to Formula (2):

(2) $$\rho =R\frac{A}{L}$$

In the formula, ρ represents the resistivity of the composite, kΩ·cm; A represents the cross-sectional area of the BC segment, cm^2^; and L represents the length of the BC segment, cm.

#### 2.3.2. Sensitivity

1. Humidity sensitivity

The samples were dried to constant weight in the vacuum oven at 45 °C. During immersion, the specimens were allowed to dry naturally and weighed at regular intervals. And the water content can be calculated by Formula (3):

(3) $$i=[(m_{i}\;-\;m_{0})/m_{0}]\;\times \;100\%$$

The composites were wrapped in plastic films and placed in a humidity chamber to prevent moisture from evaporating. The selected test points were 0%, 1%, 3%, 5%, 7%, and 9%. The resistivities of the specimens were measured at these different moisture levels.

In the test of humidity-sensitive performance, the fractional change in resistivity (FCR) can be expressed as Formula (4):

(4) $$\Delta \rho /\rho =\frac{\rho \;-\;\rho_{0}}{\rho_{0}}$$

In the formula, Δρ/ρ represents the FCR of the composite, %; ρ represents the resistivity of the composite with different water contents, kΩ·cm; and $\rho_{0}$ represents the resistivity of the composite after drying, kΩ·cm.

2. Temperature sensitivity

Before the test, the samples were also dried to constant weight. The specimens were wrapped with heat-shrinkable films to prevent moisture and put in a water bath to test the resistivities of the specimens under different temperature gradients. The heating interval was 5 °C, and the test range was 5 °C to 70 °C. The voltage and current under each temperature gradient were measured to calculate the corresponding resistance value.

The FCR was used to reflect its sensitive characteristics. The calculation formula is as follows:

(5) $$\Delta \rho /\rho =\frac{\rho \;-\;\rho_{0}}{\rho_{0}}$$

In the formula, Δρ/ρ represents the FCR of the specimen, %; ρ represents the resistivity of the composite at different temperatures, kΩ·cm; and $\rho_{0}$ represents the composite resistivity at 20 °C in the first cycle, kΩ·cm

3. Piezoresistive property

The specimens were also dried completely in a drying oven. After that, they were coated with waterproof paint to isolate the moisture. The specimens were placed on a universal testing machine at a room temperature of 20 °C. After the current was stable, the test started. The data of this test were collected by computer.

In the test of pressure-sensitive performance, the FCR can be expressed as Formula (6):

(6) $$\Delta \rho /\rho =\frac{\rho \;-\;\rho_{0}}{\rho_{0}}$$

In the formula, Δρ/ρ represents the FCR of the composite, %; ρ represents the resistivity of the composite under different loads, kΩ·cm; and $\rho_{0}$ represents the resistivity of the composite without load, kΩ·cm.

#### 2.3.3. Microscopic Characterization

This study adopted Scanning Electron Microscopy (SEM, Hitachi Regulus 8100, Japan) to investigate the morphology of CNT-CFs. X-ray Photoelectron Spectroscopy (XPS, Thermo Scientific K-Alpha, Waltham, MA, USA) was utilized to analyze the feasibility of this method.

## 3. Results and Discussion

### 3.1. Characterization of CNT-CFs

**Figure 3:**
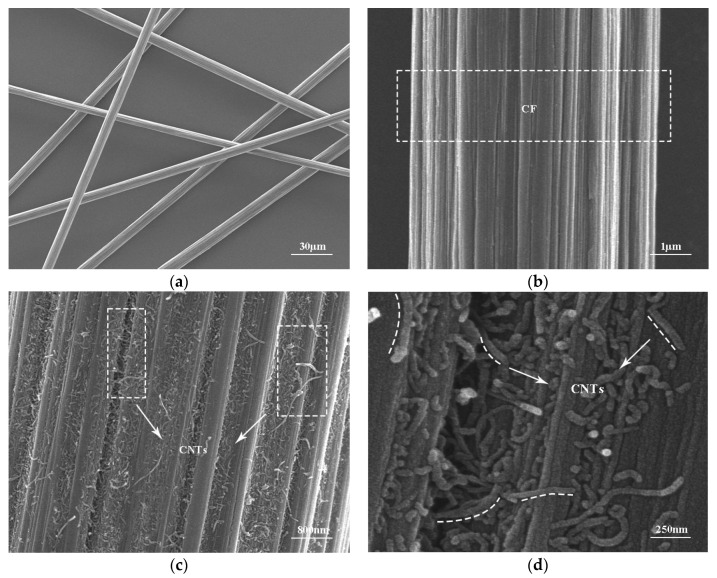
SEM images of CFs and CNT-CFs: (**a**) CFs, (**b**) enlarged surface of CFs, (**c**) CNT-CFs, and (**d**) enlarged surface of CNT-CFs.

In Figure 3a,b, it is evident that the surface of CFs was relatively smooth, featuring grooves aligned parallel to the fiber axis. This characteristic contributes to the limited strength enhancement when CFs are incorporated into the cement matrix because the connection between CFs and the matrix is not strong. In Figure 3c, it can be observed that the fibrous CNTs uniformly covered the surface of CNT-CFs. This structure increased the roughness of the fiber surface compared to unmodified CFs [38]. Furthermore, at higher magnification (Figure 3d), numerous CNTs could be seen uniformly grafted onto the surface of CFs. And the CNTs formed network structures at the groove defects. This structure could enhance the anchor between fibers and the matrix. Compared to the grafting techniques in other studies [33,35,39], the grafting technique used in this study had a considerable number of CNTs within the field of view (Figure 3c,d).

**Figure 4:**
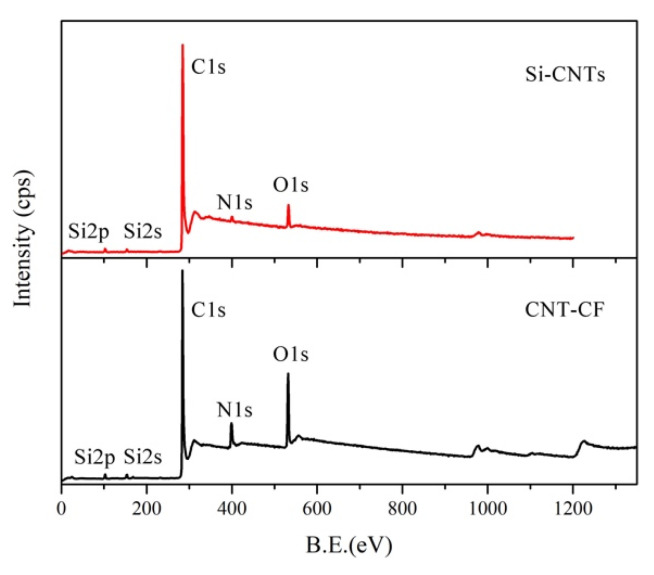
XPS of silane-modified CNTs and CNT-CFs.

To further illustrate the reliability of this grafting method, XPS was applied to characterize the structure of CNT-CFs. The test results of Si-CNTs and CNT-CFs are presented in Figure 4. It is evident in the figure that peaks corresponding to Si-2p and N1s were clearly observable. The Si and N elements were from KH550. KH550 and CNT-OH were linked by Si-O-C bonds according to Figure 1. Since the CNT-CFs were derived from the reaction between Si-CNTs and O-CFs, the distinct observation of Si-2p and N1s peaks indicated the successful preparation of CNT-CFs. The N1s content reached 8.4%, while silicon (Si2p) content attained 3.4%.

**Figure 5:**
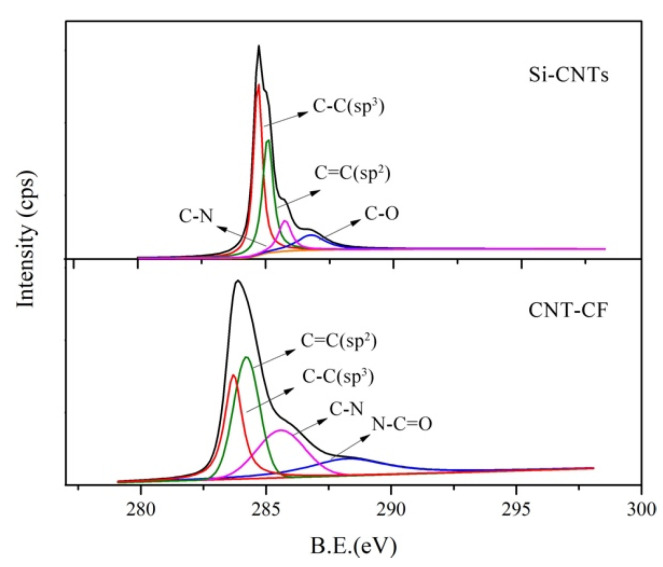
Fitting curves of surface functional groups, C1s, of silane-modified CNTs and CNT-CFs.

To further investigate the structure changes in materials before and after grafting, the C1s peak of Si-CNTs and CNT-CFs were subjected to fitting analysis. Split-peak fitting could exclude interference from other elements, thus enhancing the credibility of previous arguments. As shown in Figure 5, the C1s peak at 284.4 eV for Si-CNTs was primarily attributed to C-C bonds. After grafting, a similar peak appeared near 284.4 eV. And a new peak emerged at 288.2 eV corresponding to N-C=O, indicating that chemical interactions occurred between -NH2 groups in Si-CNTs and -COOH groups in O-CFs. The N-C=O group was not present in the structures of O-CFs and Si-CNTs.

Compared to the studies of Liu et al. [35] and Abdelghani Laachachi et al. [39], the grafted fibers obtained in this study had a greater steric effect. As a result, the fibers may have better dispersion.

**Figure 6:**
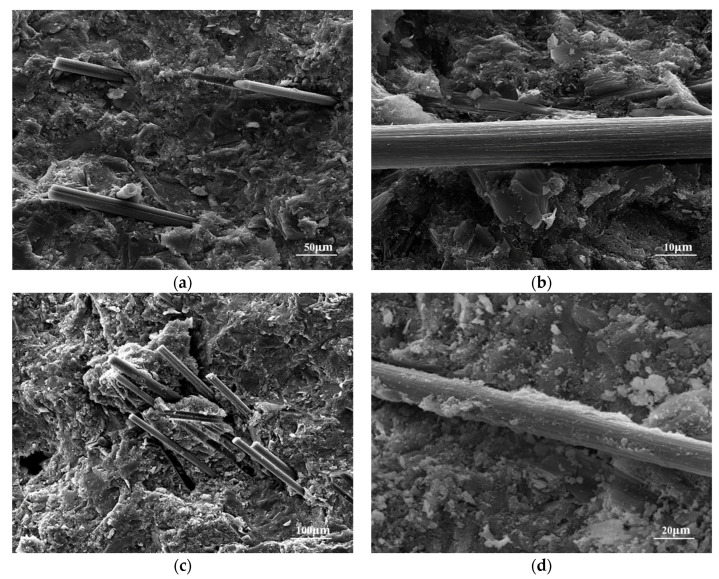
SEM images of the anchoring between fibers and the matrix: (**a**) CFs, (**b**) enlarged surface of CFs, (**c**) CNT-CFs, (**d**) and enlarged surface of CNT-CFs.

Figure 6 shows the microscopic morphology of the smart cement-based materials incorporating grafted carbon fibers and normal carbon fibers. In Figure 6a,b, the fibers were anchored in the cement matrix. At the same time, the surface of the fiber was relatively smooth. In Figure 6c,d, the grafted carbon fibers also had an anchoring effect with the cement matrix. But the surface of the fibers was rougher than the surface of normal fibers. And the anchoring between fibers and the matrix was better.

### 3.2. Test Results of Conductivity

For SCBMs, excellent conductivity is the primary determinant of their performance. Thus, this section selected the composite with the best conductivity. The performance of the SCBM selected is evaluated in the next section.

The tunneling effect conduction theory is a commonly referenced framework for discussing the conductivity of SCBMs. This theory attributes the conductivity of composites to electron transitions. The tunneling current density can be expressed by Equation (7):

(7) $$J=\frac{3\sqrt{2m\Phi }}{2s}{\left(\frac{e}{h}\right)}^{2}\;\times \;U\;\times \;e^{\left(\frac{4\pi s}{h}\sqrt{2m\Phi }\right)}$$

According to the equation, m and e are the mass and charge of a single electron, respectively; h is Planck′s constant; Φ and s represent the tunneling barrier height and barrier width, respectively; and U is the applied voltage across the barrier.

**Figure 7:**
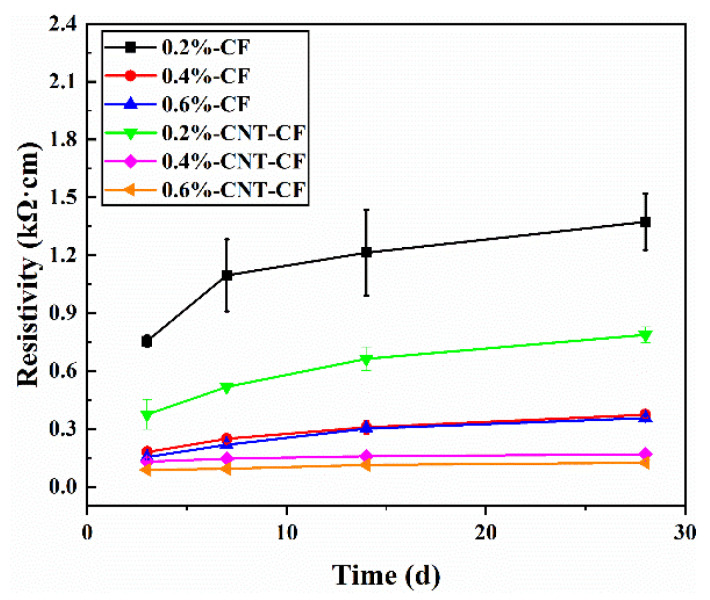
Resistivity of various samples at different ages.

Figure 7 illustrates the variation in resistivity of different samples during curing. It can be observed from the figure that the resistivities of the specimens increased continuously, exhibiting a significant change in the early stage of hydration. In contrast, in the later stage, the change in resistivity gradually diminished, leading to a stabilization in values. In the early stage, the resistivities of specimens were greatly influenced by internal water contents. As hydration progressed, the internal water content decreased gradually, resulting in a reduction in ion solution concentration. In addition, as hydration products formed, the cement matrix was denser. And the conductive path was hindered by these products.

As the CF content increased, the resistivity rapidly decreased and stabilized later. This behavior can primarily be attributed to higher CF content facilitating a more effective conductive network within the composite. When the content of the conductive filler was 0.4 wt.%, at the age of 28 d, the resistivities of the CF/CC and CNT-CF/CC were 0.373 kΩ·cm and 0.167 kΩ·cm, respectively. The resistivity decreased by 55.22%. The CNT-CF/CC had better conductivity. This may be attributed to the fact that CNT-CFs possessed a comparatively rougher surface and stronger steric effect. Consequently, the dispersion of CNT-CFs was significantly enhanced, thereby improving connectivity within the conductive networks. According to formula (7), as the barrier width (s) decreased, the conductivity was better. The addition of CNTs reduced the value of s because the distance between the conductive fillers became shorter. Therefore, the CNT-CF/CC had more conductive pathways, providing new channels for electron transitions [34].

### 3.3. Test Results of Humidity Sensitivity

**Figure 8:**
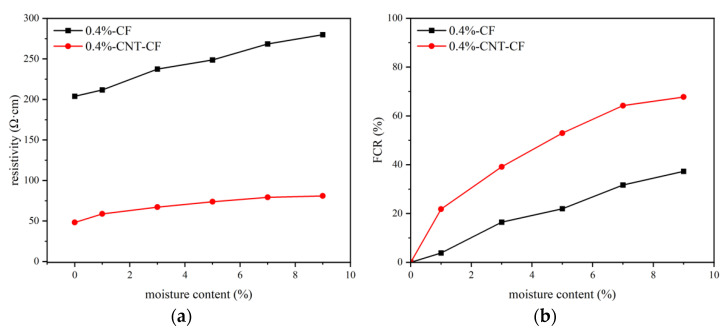
Electrical response of various samples to humidity: (**a**) resistivity, (**b**) FCR.

Figure 8 describes the electrical responses of various samples to humidity. According to the experimental results in Section 3.2, the content of conductive filler was determined at 0.4 wt.%. As shown in Figure 8a, the resistivity of samples all increased with rising moisture content. This phenomenon can be attributed to the fact that as moisture content increased, some conductive pathways formed by conductive fillers were obstructed by water [40].

In Figure 8b, it is evident that the CNT-CF/CC exhibited greater sensitivity to humidity compared to the CFs/CC. When moisture contents were at 1%, 3%, 5%, 7%, and 9%, for the CNT-CF/CC, there were corresponding increases in resistivity of 21.80%, 39.14%, 52.92%, 64.19%, and 67.79% relative to dry conditions. But for CFs/CC, there were corresponding increases in resistivity of 3.81%, 16.42%, 21.94%, 31.66%, and 37.25% relative to dry conditions. The incorporation of CNTs broadened the conductive network. As moisture content rose, more conductive pathways were blocked [41].

### 3.4. Test Results of Temperature Sensitivity

#### 3.4.1. The Conductivity Variation in Samples with Temperature

**Figure 9:**
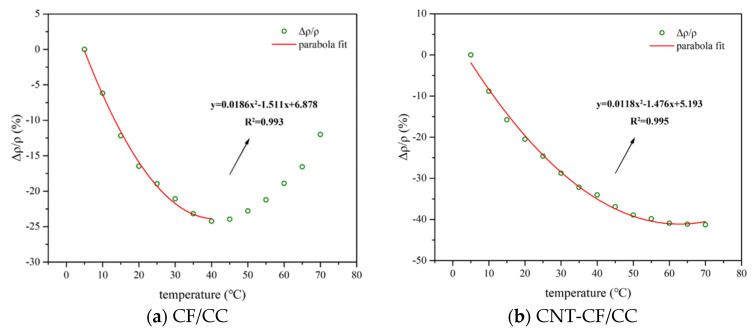
Fitting curves of electrical properties of different samples with temperature.

Figure 9 depicts the correlation between the variation in resistivity of various samples with temperature. The test range was 5–70 °C. In the figure, both the CFs/CC and CNT-CF/CC exhibited distinct trends. In Figure 9a, it can be observed that with increasing temperature, the resistivity of the CFs/CC initially decreased and increased later. This behavior is attributed to two different response mechanisms: the Positive Temperature Coefficient (PTC) effect and the Negative Temperature Coefficient (NTC) effect [42,43].

As the temperature rose, more electrons gained energy and were excited to become charge carriers [41,44], thereby enhancing the conductivity of the composite. This phenomenon is the NTC effect. Conversely, the PTC effect primarily arises from thermal expansion stress leading to cracking. The stress increased the distance between CFs and disrupted some conductive pathways [45]. In the early stages of heating, NTC effects dominated; however, as temperature continued to rise, PTC effects took precedence. These mechanisms interacted with each other resulting in a transition point at approximately 40 °C for the CFs/CC.

In contrast, Figure 9b shows that as temperature increased, the resistivity of the specimens consistently decreased. The FCR of the composite with the CNT-CFs was found to be greater at each temperature. For the CNT-CF/CC, the transition point was over 70 °C. On the one hand, as the temperature increased, more electrons in the CNTs were activated to participate in conduction, thereby enhancing the conductivity of the specimen. Because of that, the CNT-CF/CC had a larger FCR value at each temperature. On the other hand, the CNT-CFs had a rougher surface, which resulted in a tighter bond between the CFs and the matrix. The thermal expansion stress was mitigated. Because of that, the CNT-CF/CC had a higher transition temperature. In most of the studies, the change in resistivity with temperature conformed to the Arrhenius relationship [46]. However, this research found that there was an obvious turning point during the heating process. Both PTC and NTC effects affected the change in resistivity with temperature at the same time. In addition, the test results obtained were very similar to the quadratic function in geometry. When the experimental results were fitted by a quadratic function, the fitting effect was good. Compared to the initial temperature (5 °C), the FCR of the CNT-CF/CC could reach up to 41.29%, with a correlation coefficient of 0.995. The FCR of the ordinary specimen was up to 24.22%, and the coefficient reached 0.993.

#### 3.4.2. Effect of Temperature Cycling on the Conductivity of Different Samples

**Figure 10:**
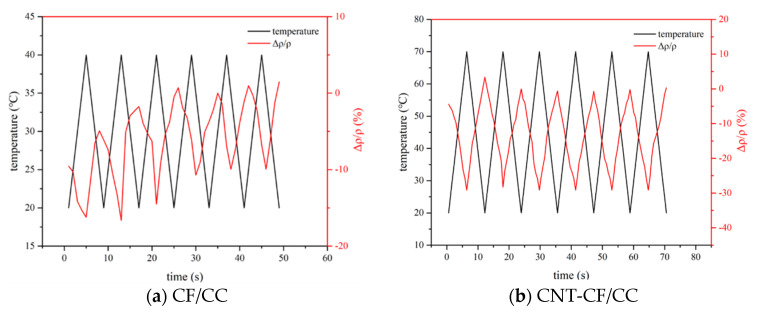
The FCR of different samples under temperature cycling

Figure 10 illustrates the variation in resistivity of various samples under temperature cycling. The peak values of the cycling temperatures corresponded to the transition points of the specimens at 40 °C and 70° C, respectively. The initial temperature was set at room temperature (20 °C).

As shown in Figure 10, the FCR of the specimens exhibited a certain cyclical pattern with fluctuations in temperature. Notably, a hysteresis phenomenon was observed. The resistivity during cooling exceeded that observed during heating. On the one hand, as the temperature decreased, charge carriers lost energy and reverted to low-energy electrons, resulting in increased resistivity. On the other hand, thermal expansion induced by rising temperatures did not fully recover during cooling phases. The CNT-CF/CC displayed a milder hysteresis effect. This improvement was primarily due to grafted CNTs enhancing the bonding between CFs and the matrix. When the temperature was 70 °C, the resistivity of the CNT-CF/CC stabilized around 28%. During the entire process, the CNT-CF/CC demonstrated advantageous temperature sensitivity and consistency.

### 3.5. Test Results of the Piezoresistive Property

#### 3.5.1. Effect of Monotonic Loading on the Conductivity of Various Samples

**Figure 11:**
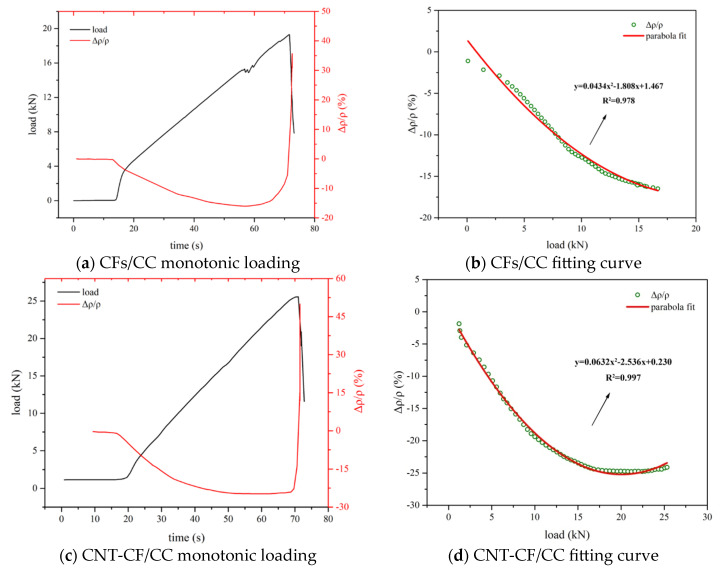
Piezoresistivity of various samples under monotonic loading.

Figure 11 demonstrates the impact of monotonic loading on the electrical performance of various samples. In Figure 11a,c, it can be observed that the failure load for the CNT-CF/CC reached 25 kN, and the failure load of the CFs/CC was 19 kN. The CNT-CF/CC had better mechanical properties. This indicated that the surface of CNT-CFs was rougher, allowing them to anchor more closely within the matrix [47,48]. The grafted carbon fibers in the study of Cui et al. [33] had less effect on the strength of the composites. However, the grafted fibers in the study of Liu et al. [35] could effectively improve the mechanical properties of the materials. Combined with the results in this study, it may indicate that the appropriate steric effect had a positive effect on the mechanical properties of the composites.

During the process of monotonic loading, the electrical response of the specimens to the applied load can generally be categorized into three distinct stages: the elastic stage, the crack development stage, and the failure stage [49].

In the initial stage, the specimen exhibited elastic response characteristics. Internal compressive stress developed within the specimen, leading to contact between conductive fillers and a subsequent reduction in resistivity. As the load increases, internal cracks gradually form and propagate within the specimen, affecting the interconnection of conductive fillers. Meanwhile, behaviors from the first stage continued to persist, resulting in relatively stable changes in resistivity. As the load further escalated towards failure, this point represented the maximum load. At this point, multiple cracks developed within the specimen; as a result, considerable disruption arose in the connectivity of the conductive fillers.

As indicated in Figure 11b,d, the specimens demonstrated a quadratic relationship between load and the FCR. In the research of Thanyarat Buasiri et al. [46] and Zhang et al. [50], the relationship between the FCR and the load conformed to a quadratic function. They also proved this inference mathematically. On the one hand, the pressure reduced the distance between conductive fillers, leading to a decrease in resistivity. On the other hand, pressure could also induce cracks within the material, disrupting the conductive network and resulting in an increase in resistivity. Both of the two mechanisms coexist simultaneously.

And the relationship between load and FCR was nonlinear. For the CNT-CF/CC, the maximum FCR was 24.77%, with a correlation coefficient reaching as high as 0.997. For the CF/CC, the maximum FCR was 16%, with a correlation coefficient reaching as high as 0.978.

#### 3.5.2. Effect of Cyclic Loading on the Conductivity of Various Samples

**Figure 12:**
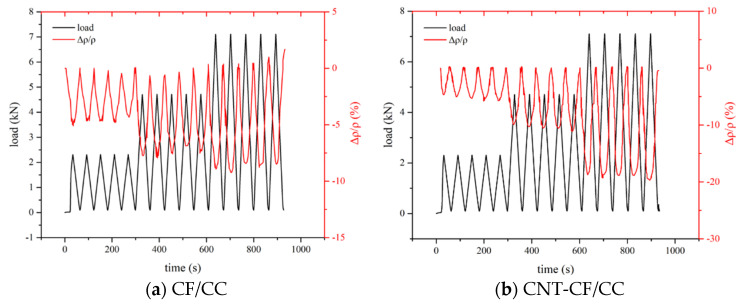
Piezoresistivity of various samples under cycle loading.

Figure 12 illustrates the piezoresistivity of different samples under cyclic loading. It can be observed from the figure that as the amplitude increased, the FCR of the specimens also rose. The greater the load, the higher the probability of overlapping among conductive fillers, resulting in an increase in the FCR. In Figure 11, it is evident that during cycling, the CNT-CF/CC exhibited maximum FCR values of approximately 7.66%, 13.67%, and 20.03% when subjected to peak cyclic loads at 15%, 30%, and 45% of failure load, respectively. But for the CFs/CC, the maximum FCR was about 4.67%, 7.56%, and 8.49% at the same load, respectively. Compared to CFs/CC, CNT-CF/CC demonstrated superior sensitivity. This enhanced sensitivity can primarily be attributed to the incorporation of CNTs, which provided more conductive pathways within the CNT-CF/CC. Additionally, CNT-CFs possessed a larger steric hindrance effect, thereby strengthening the tunneling effect within the CNT-CF/CC. Compared with the result in the study of Liu et al. [34], the curve was more stable and the FCR value was higher at each load. The result reflected that an appropriate steric effect was beneficial for improving the sensitivity of smart cementitious materials.

In order to better measure the sensing performance of the two composites, stress sensitivity (SES) was introduced [51]. Its value is shown in Equation (8):

(8) $$\mathrm{SES}=\frac{{-\mathrm{FCR}}_{\max}}{\sigma }$$

where σ is the amplitude of the measured compressive stress during the cyclic loading test, MPa; ${\mathrm{FCR}}_{\max}$ is the corresponding amplitude of the FCR during cyclic loading, %; and σ was obtained by Equation as follows (9):

(9) $$\sigma =\frac{F}{A}$$

F is the corresponding external load; N. A represents the pressure cross-sectional area, m^2^

This paper calculated the SES of the two composites at each amplitude, and the values are shown in Table 5. In this way, this study can better illustrate the effectiveness of the method. It can be seen in Table 5 that the SES of the CNT-CF/CC under each load was higher.

## 4. Discussion

In this section, this paper systematically summarizes the results obtained from previous studies to enhance the overall structure of this work. Before describing the mechanism, it is necessary to compare this study with previous studies. However, a large number of studies added conductive fillers directly. This article focused more on the preparation process, so it is improper to compare with these studies. This article compared the improvements of the oriented technique and grafting technique on the sensing performance because the oriented technique also improves the preparation process to obtain the expected results. In order to illustrate the influence of the steric effect, this article compared the performance of cementitious composite incorporating grafted carbon fibers obtained by other grafting methods. The results are shown in Table 6.

Table 6 shows that the oriented technique had the highest sensitivity, but its improvement in performance was limited. The grafting technique used in this paper had a particularly obvious improvement in sensitivity. It shows that the fiber structure obtained by the grafting technique was good for the dispersion of fibers, greatly improving the performance of the composite. The lower sensitivity may be due to the shorter spacing between conductive fillers. The appropriate spacing increased the amplitude of the FCR in the loading process. In addition, the performance of smart cement-based materials was greatly affected by the cement matrix, so it was more meaningful to compare the improvement in performance.

**Figure 13:**
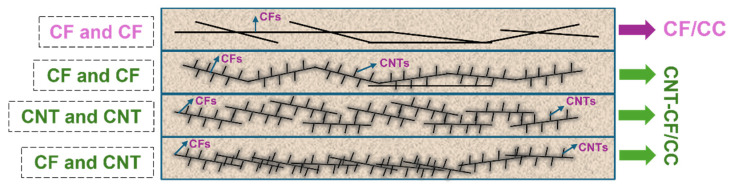
The overlaps between conductive fillers in the composite.

After comparison, we can conclude that the primary factors influencing the conductivity and sensitivity of the composites include two aspects. Firstly, because of the introduction of CNTs, there are more options for conductive fillers to contact in the CNT-CF/CC. As illustrated in Figure 13, within the CNT-CF/CC, the overlap configurations of conductive fillers include overlaps between the CNTs, overlaps between the CFs, and overlaps between the CNTs and CFs. In contrast, within the CFs/CC, the overlap configuration is limited to overlaps between the CFs. The overlap of conductive fillers significantly affects the conductivity of SCBMs. And it can be concluded that the CNT-CF/CC exhibits superior conductivity. Furthermore, due to the diverse overlapping configurations of conductive fillers within the CNT-CF/CC, its internal conductive network is also more stable. Consequently, during temperature cycling and pressure cycling tests, the CNT-CF/CC demonstrated more stable cyclic curves. However, such assertions seem to contradict some experimental findings obtained in the previous section. For instance, the sensitivity to the environmental change in the CNT-CF/CC was higher. This discrepancy primarily arises from the stronger conductivity of the CNT-CF/CC, leading to a huge response to external environmental changes. Additionally, during pressure and temperature cycling tests, the robust conductive network of the CNT-CF/CC exhibited significant recovery capability resulting in more regular curves. In addition, the surface of the CNT-CFs features numerous grafted CNTs. However, the surface of conventional CFs is smoother. The incorporation of grafted CNTs enhances the bonding strength between the CNT-CFs and the cement matrix. As a result, the CNT-CF/CC displays greater performance stability.

## 5. Conclusions

This study adopted a chemical grafting method to attach CNTs onto the surface of CFs using a coupling agent as an intermediate. The feasibility of this approach was demonstrated through SEM analysis and XPS analysis. Furthermore, CNT-CFs were incorporated into the cement matrix to fabricate the CNT-CF/CC. Compared with the CF/CC, the variations in conductivity and sensitivity performance were investigated. The conclusions drawn from this research are as follows:

(1) Characterization of the CNT-CF through SEM reveals that compared to untreated CFs, the surface of the CNT-CFs was rougher. This structural feature has profound implications for the properties of the CNT-CF/CC.
(2) In the test results of XPS, characteristic functional groups introduced by coupling agents on the CNTs could be seen in the spectrum, confirming the effectiveness of the grafting method. Analysis of chemical bonds within the CNT-CFs showed that strong chemical bonding connected the CNTs and CFs, successfully.
(3) The resistivity of the CNT-CF/CC increased continuously during curing. Compared to the CF/CC, there is a significant improvement in conductivity for the CNT-CF/CC. The incorporation of CNTs provided additional conductive pathways, thereby improving its electrical properties.
(4) Compared to the CF/CC, the CNT-CF/CC demonstrated superior moisture sensitivity. The resistivity of specimens increased with rising moisture content. Water infiltration adversely affected the conductivity of the composite by disrupting conductive pathway formation.
(5) Compared to the CF/CC, the CNT-CF/CC exhibited enhanced thermal sensitivity as well. The CNT-CF/CC showed better measurement range and performance stability. The bond between the CNT-CF and the cement matrix was tighter, resulting in improved conduction stability in the test.
(6) Compared to the CF/CC, the CNT-CF/CC also displayed superior piezoresistivity. Under monotonic loading, both the failure load and FCR values of the CNT-CF/CC were greater. Under cyclic loading, the curve stability remained robust, and piezoresistive performance was more stable. The anchoring connection between the CNT-CFs and the cement matrix was identified as a primary factor.

In general, CNT-CFs can further achieve the aim of applying SCBMs in real engineering. Compared to the previous studies, the CNT-CF prepared by the coupling method was easier to synthesize and exhibited clearer steric effects. These advantages made the performance of the CNT-CF/CC sensitive and stable. Compared to other methods, the method adopted in this paper is easy to scale up. Large-scale production is more conducive to ensuring the stability of grafted fiber performance. At the same time, grafting techniques are easier to combine with other methods. This is exactly the significance of this study.

## Figures and Tables

**Table 1:** Basic physical performance of the cement.

Density	Standard Consistency Water Consumption	Specific Surface Area	Condensation Time		Flexural Strength		Compressive Strength	
			Initial	Final	3 d	28 d	3 d	28 d
3.2 g/cm^3^	0.273	350 m^2^/kg	182 min	225 min	5.3 Mpa	9.4 Mpa	22.7 Mpa	50.2 Mpa

**Table 2:** Basic physical properties of the CFs.

Length	Diameter	Tensile Strength	Tensile Modulus	Density	Carbon Content	Resistivity
5 mm	7 μm	4.53 Gpa	230 Gpa	1.79 g/cm^3^	93%	1.6 μΩ·cm

**Table 3:** Basic physical properties of the CNTs.

Purity (wt%)	Outer Diameter (nm)	Length (μm)	Specific Surface Area (m^2^/g)	Ash Content (wt.%)	Conductivity (s/cm)	-OH Content (wt.%)
>95	5–15	0.5–2	0.27	~2.1	>100	5.58 wt.%

**Table 4:** Raw material mix proportion (wt.%).

Sample	Cement (g)	Water (g)	CFs (wt.%)	CNT-CFs (wt.%)	Dispersant-CF (wt.%)	Defoamer (g)
CF/CC	200	80	0.2	0	0.2	0.26
			0.4	0	0.4	
			0.6	0	0.6	
CNT -CF/CC	200	80	0	0.2	0.2	0.26
			0	0.4	0.4	
			0	0.6	0.6	

**Table 5:** SES of different samples at variable loads.

Sample	15%- Failure Load	30%- Failure Load	45%- Failure Load	Average
CF/CC	0.65	0.53	0.40	0.53
CNT-CF/CC	0.82	0.73	0.71	0.75

**Table 6:** Properties of different composites prepared by different methods.

Sample	Best Resistivity	Best SES	Best Improvement on SES
Oriented technique [22]	about 10^4^ Ω·cm	2.36	Increase by 35%
Grafting technique [35]	about 0.4 kΩ·cm	0.22	Decrease by 27.76%
Grafting technique (this article)	0.12 kΩ·cm	0.82	Increase by 77.50%

## Data Availability

The original contributions presented in this study are included in the article. Further inquiries can be directed to the corresponding author.
